# Prevalence of Non-adherence to Antiepileptic Drugs in Patients With Epilepsy Presenting to Emergency With Fits

**DOI:** 10.7759/cureus.27072

**Published:** 2022-07-20

**Authors:** Shakeel A Awan, Imran Khawaja, Muhammad Babar, Faheem Khan

**Affiliations:** 1 Department of Medicine, University Hospitals of Derby and Burton, Burton, GBR; 2 Department of Internal Medicine, Ayub Teaching Hospital, Abbottabad, PAK; 3 Department of Medicine, University Hospitals Birmingham NHS Foundation Trust, Birmingham, GBR; 4 Department of Acute Medicine, Queen's Hospital Burton, Burton, GBR

**Keywords:** adherence, seizures, epilepsy, compliance, central nervous system, antiepileptic, aed

## Abstract

Background

Epilepsy is considered when a patient has at least two unprovoked seizures that occurred more than a day apart. Seizure control depends upon several factors, including adequate treatment and its dosage, patients' daily activities, and adherence to antiepileptic medications. The study aimed to assess the rate of adherence to antiepileptic drugs (AED) in patients with epilepsy.

Methodology

A cross-sectional study was conducted at the Department of Neurology, Ayub Teaching Hospital, Abbottabad, Pakistan, between November 2019 and October 2020. All participants who presented to the emergency room with complaints of seizures, had a known diagnosis of epilepsy, aged above 18 years, with no cognitive dysfunction or severe psychiatric disorders were included in the study. Patients with other neurological disabilities (brain tumors, cerebral palsy, neuromuscular disorder) or severe psychotic episodes and those with undiagnosed cases of epilepsy were excluded from the study. A predefined proforma was used to assess the level of adherence and non-adherence among patients and they were then divided into their respective groups.

Results

A total of 150 participants were included in the study. Of patients, 110 were adherent to AED treatment while 40 were non-compliant. It was found that the most frequent cause of non-adherence was that patients forgot their pills (72.5%). Of patients, 7.5% stopped taking the medication when symptoms were relieved. About 12.5% reported affordability to be the reason for non-adherence. The rate of poor seizure control was significantly higher in non-adherent patients as compared to adherent patients (77.5% vs. 49.1%, p = 0.001). It was also found that a greater number of non-adherent patients experienced convulsive seizures in the past year as compared to those who were adherent to their medications (p = 0.006).

Conclusion

To enhance treatment adherence, the practice of prescribing more simpler treatment regimens among physicians can result in better seizure control, as the complexity of the regimen is found to be a major challenge for adjustment of AED regimens in this regard.

## Introduction

Epilepsy is a chronic central nervous system (CNS) disorder. The global impact of epilepsy can be understood by surveying statistics regarding the neurologic disorder. According to an estimate by the World Health Organization (WHO), 50 million people are impacted by epilepsy globally [1]. About three-fourths of these people live in developing nations [1]. In developed regions of the world, the prevalence is found to be about eight per 1000 population. This establishes epilepsy as one of the most prevalent chronic neurologic disorders [2].

Proper clinical management of epilepsy requires strict adherence to prescribed medication. Unfortunately, non-adherence is common and can cause recurrence of seizures despite treatment [3]. Non-adherence to prescribed antiepileptic drugs (AED) has a grave impact on the management of this controllable disorder. This leads to a variety of adverse consequences like more frequent emergency and clinic visits with subsequent hospitalizations, physical injuries, and affected daily functioning. All these factors affect the overall cost of managing epilepsy and have a significant undesirable economic impact [4,5].

One of the most common challenges faced in epilepsy management is non-adherence to medication, which can result in serious health-related and financial outcomes [4]. It is suspected that non-adherence to the AED regimen may cause a halt in seizure control [6]. As discussed earlier, poor or non-adherence to prescribed regimens leads to numerous damaging effects on the patient and exhausts the capacity of present clinical resources [4]. The healthcare and the financial load of poor seizure control can only be countered with increased adherence. Increased adherence reduces the load upon healthcare facilities with regards to recurrent unpredictable visits to both outpatient and emergency services, and the frequency of inpatient admissions [7]. As evidenced by numerous sources in the literature, non-adherence is associated with and can be explained by four principal types of issues. These factors of non-adherence include clinician-patient dynamics, issues regarding the patient’s illness itself such as disease intensity and symptom frequency, and the ease of following the regimen; greater side effects, poorer patient-perceived control of symptoms, and numerous daily dosages all decrease adherence; and finally, patient’s personal concerns, circumstances, and social and economic knowledge and awareness affect adherence [8,9]. Identifying patients at high risk for non-adherence and following an approach targeted at their individual and specific issues will ultimately lead to better drug adherence and will reduce adverse outcomes including mortality [10,11].

Due to a dearth of knowledge on the matter and a lack of local literature, the present study was undertaken at a tertiary care center. The present research aimed to study the factors related to non-adherence to antiepileptic drugs in patients with epilepsy.

## Materials and methods

A cross-sectional study was conducted at the Department of Neurology, Ayub Teaching Hospital, Abbottabad, Pakistan, between November 2019 and October 2020. After procuring the ethical letter from the institutional review board (#IRB/NEU/4524), the data acquisition was started.

Using the non-probability convenience sampling technique, patients were recruited in the study. All individuals who presented to the outpatient department for follow-up visits or those who presented to the emergency department with complaints of seizures, and those who were diagnosed with epilepsy in the last two years were eligible to partake in the study. Patients aged above 18 years with no cognitive dysfunction or severe psychiatric disorders were included in the study. Patients with other neurological disabilities (brain tumor, neuromuscular disorder, cerebral palsy, etc.) or severe psychotic episodes, and those with undiagnosed cases of epilepsy were excluded from the study.

Medication adherence was defined as the degree to which individuals follow doctors' orders and take their medicine. Seizure control was defined as “good” in patients who had been taking medication as per doctors’ instructions and were seizure-free for the last six months. Patients with “poor” seizure control were defined as those who were not taking medication as per doctors' instructions and experienced a seizure in the last six months [11].

Prior to the patients' enrollment in the study, the significance of the study was narrated to the individuals by the researchers, and informed verbal and written consent was requested from them. A predefined proforma was used to assess the level of adherence among patients and they were then divided into their respective groups, i.e., adherent and non-adherent groups. Data collection involved the documentation of patient demographics, details of seizure occurrence, incidence and pattern, comorbid conditions and disabilities, drug regimens, perceived adherence to prescribed medications, and general well-being. Self-perceived adherence was evaluated by requiring research participants to remember missed or stopped doses in the last three months, one month, and one week.

The number of drugs prescribed, frequency of seizure episodes in the last year, depression symptoms, employment status, and handicap or any disability were documented. Statistical software Stata version 13 (StataCorp LLC, College Station, TX) was used to assess the data. A descriptive analysis of the data was carried out using t-tests and chi-square tests. The cut-off for significance level (alpha) was considered to be below 0.05. All data were presented as mean and standard deviation if the variables were non-discreet or quantitative. Discrete and categorical data were represented as frequency and proportions.

## Results

A total of 150 participants were included in the study. Of patients, 110 were adherent to AED treatment while 40 were non-adherent. The characteristics of study participants are illustrated in Table 1. The sociodemographic factors did not correlate significantly with non-adherence.

**Table 1:** Characteristics of study participants

Parameters	Total (150)	Adherent (110)	Non-adherent (40)	P-value
Gender				0.2
Male	77 (51.3%)	53 (48.2%)	24 (60%)	
Female	73 (48.7%)	57 (51.8%)	16 (40%)	
Age mean (years)	43.3 ± 11.7	44.2 ± 11.7	41.3 ± 11.8	
Age				0.272
18-19	1 (0.7%)	1 (0.9%)	0 (0%)	
20-29	21 (14%)	15 (13.6%)	6 (15%)	
30-39	32 (21.3%)	19 (17.3%)	13 (32.5%)	
40-49	48 (32%)	40 (36.4%)	8 (20%)	
50-59	34 (22.7%)	24 (21.8%)	10 (25%)	
60+	14 (9.3%)	11 (10%)	3 (7.5%)	
Marital status				0.398
Single	44 (29.3%)	32 (29.1%)	12 (30%)	
Married	87 (58%)	66 (60%)	21 (52.5%)	
Divorced	14 (9.3%)	9 (8.2%)	5 (12.5%)	
Separated	3 (2%)	1 (0.9%)	2 (5%)	
Widowed	2 (1.3%)	2 (1.8%)	0 (0%)	
Education status				0.982
No formal education	7 (4.7%)	5 (4.5%)	2 (5%)	
Primary school	1 (0.7%)	1 (0.9%)	0 (0%)	
Secondary school	56 (37.3%)	41 (37.3%)	15 (37.5%)	
College	72 (48%)	53 (48.2%)	19 (47.5%)	
Graduate	14 (9.3%)	10 (9.1%)	4 (10%)	

Upon exploring the causes of non-adherence among patients, it was found that the most frequent cause of non-adherence was that patients forgot their pills (72.5%). Of patients, 7.5% stopped taking the medication when symptoms were relieved. About 12.5% reported affordability to be the reason for non-adherence (Table 2).

**Table 2:** Causes of non-adherence among patients with epilepsy

Factors	N (%)
Forgot/did not have pills	29 (72.5%)
Side effects	4 (10%)
Cost	5 (12.5%)
Dosing	1 (2.5%)
No symptoms	3 (7.5%)

In Table 3, occurrences of seizures have been associated with non-adherence. The rate of poor seizure control was significantly higher in non-adherent patients as compared to adherent patients (77.5% vs. 49.1%, p = 0.001). One or more seizures in the past year were experienced by 62.5% of patients who were non-adherent to AED (p = 0.004) treatment and half of the non-adherent population claimed that they missed a dose just before they experienced a seizure episode. Moreover, a significantly higher number of non-adherent patients experienced convulsive seizures in the past year than those who were adherent to their medications (p = 0.006).

**Table 3:** Association between non-adherence and seizure activity

Parameters	Adherent (110)	Non-adherent (40)	P-value
Poor seizure controlled	54 (49.1%)	31 (77.5%)	0.001
One or more seizures in the past year	40 (36.4%)	25 (62.5%)	0.004
Experienced convulsive seizures in the past year	20 (18.2%)	16 (40%)	0.006
Missing dose before seizures	40 (36.4%)	20 (50%)	0.132

## Discussion

In the present study, the researchers dwelled on the contributory factors associated with non-adherence to AED. We found that the sociodemographic factors did not associate significantly with non-adherence to AED. Upon further exploration, we found that in the majority of cases, patients just forgot to take their pills on time. Only 7.5% reported that the reason they were not taking regular medication was relief from symptoms. We also found that non-adherence to AED was significantly correlated with poor seizure control and more than half of them experienced seizures in the last year. Thus, highlighting the deleterious effects of non-adherence with AED.

Some studies explore the relationship between the characteristics of AED with adherence among patients. One such study by Bautista et al. revealed that older AEDs including carbamazepine, ethosuximide, and phenobarbital, among others, superseded the new regimens such as lamotrigine, levetiracetam, and pregabalin [12].

A study conducted in Brazil by Ferrari et al. demonstrated a non-adherence rate of 66.2%, i.e., a moderate-to-low level of adherence. Men, those with complex AED regimens, those with poorly controlled seizures, and younger patients had increased rates of non-adherence. This was measured by the Morisky Green Levine Medication Adherence Scale (MGLS) [13].

Our study revealed that patients who were non-adherent to medication were more likely to have a seizure than those who took their medicine regularly. Similar findings were revealed by another study published by Manjunath et al., who found that seizure risk was approximately one-fourth percentage greater in non-adherent patients than in adherent patients (hazard ratio = 1.205, p = 0.0002) [14]. Literature has shown that patients who are non-compliant with AED treatment have a poorer prognosis. A comprehensive study comprising 33,658 patients, of whom 26% were non-adherent, revealed that non-adherence was significantly correlated with an approximately three-fold augmented risk of mortality as compared to those who were compliant with AED (hazard ratio = 3.32, 95% CI = 3.11-3.54). Furthermore, the length of non-adherence is associated with a higher incidence of emergency visits due to seizure activity among patients [15].

Non-adherence to AEDs may have serious or even lethal consequences for epileptic patients. Our study findings are in line with a Chinese study by Liu et al., in which no relationship between sociodemographic parameters and adherence to AED was found. However, adherence was significantly associated with the length of the disease (p = 0.007). The authors also revealed that the main cause of non-adherence was forgetfulness [16]. Recently, a systematic review was conducted to assess the hindrances against the non-adherence to AED among epileptic patients.

The literature review presented illustrates the widespread low adherence to AED regimens. This non-adherence leads to ineffective seizure control and significantly affects the quality of life [17]. Contributory factors to this rampant low adherence are found to be poorly controlled recently recurring seizures, preconceived notions about specific medications, mood disorders (i.e., depression and anxiety), lack of necessary social support, numerous daily doses of AED, inadequate self-administration, and an unsatisfactory therapeutic relationship with the clinician. Moreover, non-adherence to AED had a negative effect on the quality of life due to poor seizure control [17].

Strategies designed to improve treatment adherence should address the issue of forgetting to take the pills on time [3]. Prescription practices that address the factors affecting adherence are essential. Manageable and uncomplicated regimens significantly increase adherence. While reducing the intricacy of treatment plans is challenging for the clinician, it may yet improve adherence in non-compliant populations.

The present study contributes significantly to the local literature as the study offers insights into the association between a modifiable behavior (adherence to medication) and seizure control in patients with epilepsy. However, there are some limitations to the study. For instance, the use of a non-probability convenience sampling technique to recruit participants in the study could have led to selection bias. Therefore, future studies with a comprehensive study design are needed to explore the impact of adherence on patient outcomes.

## Conclusions

We found that patients who were non-adherent with their antiepileptic medications had poorer seizure control and more than half of them experienced seizures in the last year. Thus, the study highlights the deleterious effects of non-adherence with antiepileptic medications. Adequate symptom control in patients diagnosed with epilepsy requires patient-centered treatment plans and thorough patient counseling regarding the disease process and management to foster better self-administration practices. This will result in an improved quality of life and subsequently reduce the economic burden.
